# Maximal aerobic capacity exercise testing protocols for elderly individuals in the era of COVID-19

**DOI:** 10.1007/s40520-021-01858-3

**Published:** 2021-04-21

**Authors:** Massimo Venturelli, Emiliano Cè, Mara Paneroni, Marco Guazzi, Giuseppe Lippi, Antonio Paoli, Carlo Baldari, Federico Schena, Fabio Esposito

**Affiliations:** 1 Department of Neuroscience, Biomedicine and Movement Sciences, University of Verona, Via Casorati 43, Verona, Italy; 2 Department of Internal Medicine, University of Utah, Salt Lake City, Utah USA; 3 Department of Biomedical Sciences for Health (SCIBIS), University of Milan, Milan, Italy; 4 IRCCS Galeazzi Orthopedic Institute, Via Riccardo Galeazzi, 4, 20161 Milan, Italy; 5 Respiratory Rehabilitation, Istituti Clinici Scientifici Maugeri IRCCS, Lumezzane, Brescia Italy; 6 Cardiology Department, University of Milano, Policlinico San Donato, Milano, Italy; 7 IRCCS, Policlinico San Donato, Milano, Italy; 8 Section of Clinical Biochemistry, Department of Life and Reproduction Sciences, University of Verona, Verona, Italy; 9 Department of Biomedical Sciences, University of Padova, Padua, Italy; 10 eCampus University Novedrate (CO), Novedrate, Italy; 11 Centro di Ricerca “Sport, Montagna e Salute”, Via del Ben 5b, Rovereto, Italy

**Keywords:** Exercise test, Virus, COVID-19, Cardiovascular function, Pulmonary function, Elderly

## Introduction

Monitoring the clinical evolving stages and cardiopulmonary sequelae, in terms of organ injury and functional response, of elderly individuals infected with SARS-CoV-2 is of utmost importance for our times, placing gas exchange and functional capacity evaluation in the forefront of the clinical decision-making process, i.e., for staging, prognostication and for establishing the most appropriate therapeutic strategies tailored for the elderly population that is the most impacted by this pandemic. From a geriatric point of view, the assessment of clinical and functional outcomes, such as mobility of the elderly individuals, their daily energy expenditure and other indices of physical function and quality of life coupled with the determination of the maximal aerobic capacity, one of the most important predictors of independence, offers a multidimensional evaluation-tool useful for monitoring the COVID-19 effects in the elderly population. Historically, cardiopulmonary exercise test (CPET) evaluation has multifold goals, such as providing a thorough and objective definition of physical limitation and garnering information on how interventions may impact the limiting steps in the symptoms cascade and along the natural course of disease.

This information and the step-by-step analyses on differential diagnosis of organ-driven origin of symptoms are generally accomplished by performing preliminary static pulmonary function tests (spirometry) and alveolar gas diffusion for carbon monoxide (DLco) evaluation.

Most of the evidence so far accumulating on COVID-19 patients suggests that: (a) the lung organ injury is sustained, persistent, expectedly irreversible in many patients [1] and is predicted to become the main clinical issue on the long term; (b) cardiovascular comorbidities are highly represented, and a very frequent background for associated complications; (c) myocarditis or acute coronary syndrome may complicate the acute phase. Irrespective of documented myocarditis, the values of the convectional biomarkers of myocardial injury, especially cardiac troponins, may considerably increase. These COVID-19 complications may occur in previously asymptomatic or symptomatic subjects, without or with lung and/or cardiac pre-existing diseases. Specifically, subjects with recognized left/right heart disease, ischemic heart disease, pulmonary hypertension, lung fibrosis, pulmonary embolism, venous thromboembolism, diffusive impairment, restrictive lung function pattern and effort-related SpO_2_ desaturation need to be accounted before starting exercise testing. It is important to note that the detrimental effects of aging on the cardiovascular and pulmonary systems are exacerbated by the COVID-19 sequels.

Based on previous long-term observations of patients with COVID-19, those needing intensive care at admission displayed a reduction in lung function spirometry FVC, TLC and 23.7% decline in DLco compared to patients who did not need intensive care unit (ICU) admission [2].

Based on this evidence and assuming that at least one out three of elderly individuals with COVID-19 has been admitted to the ICU, the application and the derived information by lung function test, gas exchange and CPET will be foundational and straightforward in progressive evaluation. Moving forward from the acute clinical scenarios, the determination of COVID-19-induced maximal aerobic capacity limitations, coupled with the determination of potential functional and physical impairments are imperative before starting specific rehabilitation strategies in the elderly individuals with long term cardiovascular and pulmonary impairments.

## Clinical phases treatments of COVID-19

### Early infection, 1st phase

The virus is present in the upper airways. An initial immune response develops, with production of antibodies and priming of cytotoxic lymphocytes, which is then accompanied by a sustained inflammatory reaction. In this phase, usual treatments are based on drugs with mixed antiviral and anti-inflammatory activity and antibacterial drug with minimal anti-viral action. Even though the physical function of the elderly patients in this phase of the COVID-19 is not extremely compromised, our recommendation is to undertake the protocols for the CPET after the natural ending of the infection, when patients muscle weakness and fatigue are not exacerbated by the disuses.

### Pulmonary-proliferative 2nd phase

In this phase, the virus migrates into the lower respiratory tract (i.e., lungs). This phase can be characterized by low peripheral oxygen saturation (SpO_2_ < 95%). Endothelial and initial cardiac damage are also possible. At this stage, hospitalization in semi-intensive care units may be necessary for administration of standard oxygen therapy. Patients who fail to ameliorate with standard oxygen therapy may be relocated to advanced oxygen and mechanical ventilation. Frequently, frail elderly patients may also have increased work of breathing and challenging breathing, which could be managed via non-invasive ventilation such as continuous positive airway pressure (CPAP) or bi-level positive airway pressure (BiPAP). Patients with persistent hypoxic failure usually undergo prone ventilation and, when the respiratory failure is severe, intubation and forced mechanical ventilation. Due to the severity of the pulmonary, cardiovascular dysfunction and disuses-induced muscle weakness no maximal exercise tests are recommended in this phase of the COVID-19 infection.

### Pulmonary-hyper-inflammatory 3rd phase

This phase is characterized by systemic symptoms with multi-organ involvement and sometimes cardiac failure. Specific treatments in this phase are needed, including corticosteroids, human immunoglobulins, inhibitors of IL-6, IL-2, and JAK2 receptor. This phase typically encompasses hospitalization in ICU or respiratory ICU. Similar to the 2nd phase, as lung dysfunction progresses, oxygen-therapy shall be increased until non-invasive mechanical ventilation is required. Due to the severity of the pulmonary, cardiovascular dysfunction and disuses-induced muscle weakness no maximal exercise tests are recommended in this phase of the COVID-19 infection.

### Vasculitic-thrombotic 4th phase

This phase is characterized by massive endothelial impairment, pro-thrombotic and hypercoagulable state, with frequently associated pulmonary thrombosis and hypertension. Specific drug treatments in this phase encompass anticoagulants (especially heparin) or phosphodiesterase inhibitors releasing nitric oxide for managing pulmonary hypertension. Due to the severity of the pulmonary dysfunction and disuses-induced muscle weakness, no maximal exercise tests are recommended for elderly patients this phase of the COVID-19 infection.

## Specific exercise testing procedure

**Fig. 1:**
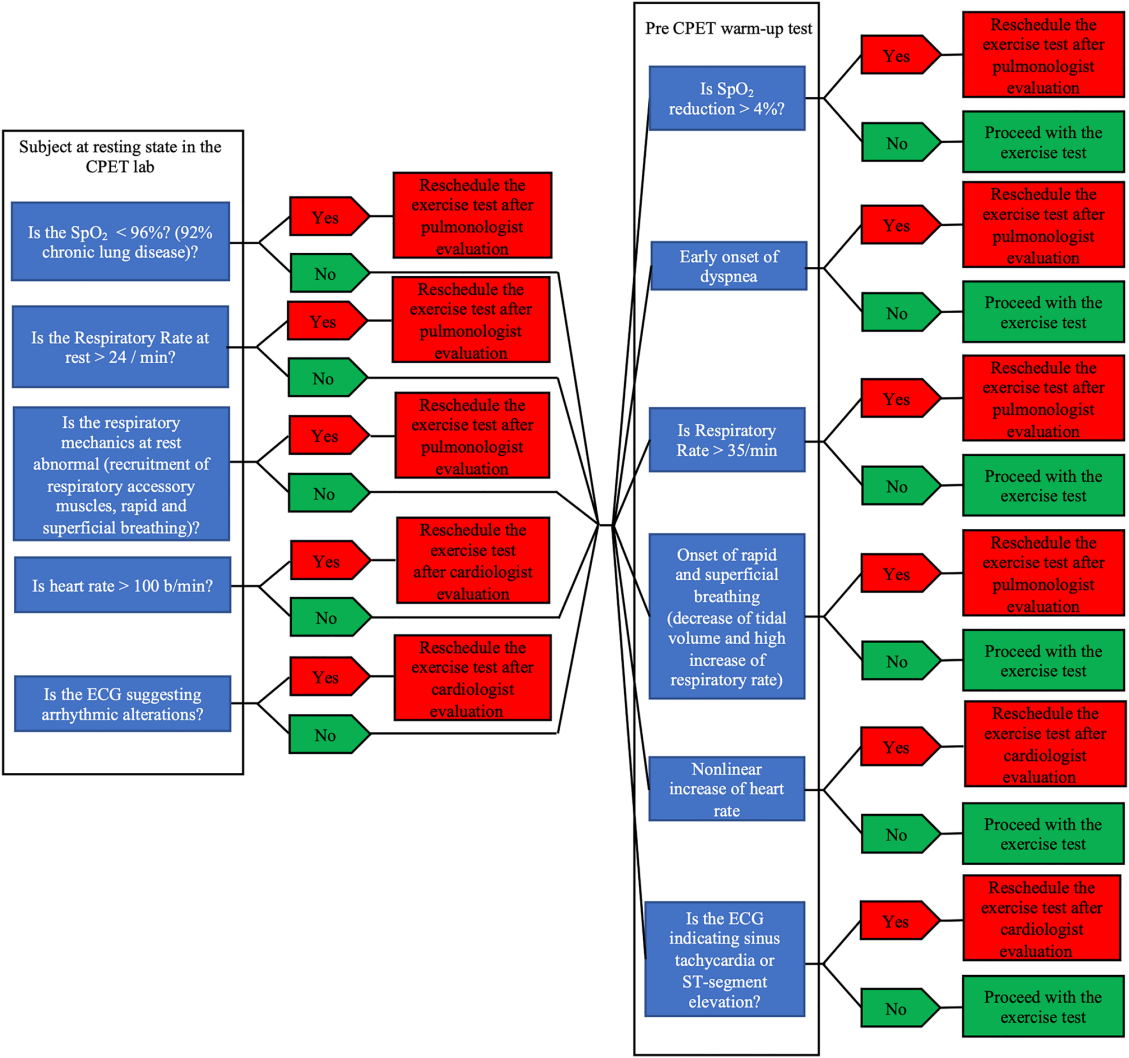
Respiratory and cardiovascular alert signs in elderly population in the era of COVID-19. CPET: cardio-pulmonary exercise test; SpO_2_: blood oxygen saturation, b/min: beats for minute, ECG: electrocardiography

Due to involvement of pulmonary and cardiovascular systems during exercise testing, and as previously discussed, the impact of both aging and COVID-19 on pulmonary and cardiovascular function, specific attention needs to be focused on respiratory and cardiovascular alert signs before and during exercise testing. It is important to note that the indirect effect of social distancing and the clinical procedures (bed confinement) adopted for the elderly individuals with COVID-19 severely impacted the level of leisure physical activity, exacerbating the skeletal muscle weakness and fatigue due to the disuse of locomotor muscles. This severe peripheral limitation is a limit that need to be accounted for the precise assessment of pulmonary of cardiovascular limitations during high intensity exercise. Therefore, before starting a maximal aerobic test such as CPET, two aspects need to be accounted:

- In case the elderly individual presents a reduced level of physical activity, muscle weakness and fatigue we recommend postponing the CPET scheduling after geriatric and physiatrist assessment.
- Low-level activity (e.g., plane walking or cycling for at least 5 min) is recommended, with continuously monitoring SpO_2_ and recording electrocardiogram (ECG) before and after the test (or via telemetry). If effort-related desaturation occurs, detected (SpO_2_ reduction ≥ 4%), and abnormal ECG changes (arrhythmic alteration such as atrioventricular block, sinus tachycardia or ST-segment elevation) are detected, we recommend postponing the CPET and scheduling after cardiological or pulmonary assessment. The detailed respiratory and cardiovascular alert-signs before and during exercise testing for elderly individuals are described in Fig. 1.

## Exercise testing protocols for elderly individuals in the era of COVID-19

Elderly population, especially is case of a sedentary lifestyle, as well as for elderly individuals with moderate to severe cardiovascular or pulmonary diseases, moderate testing protocols should be considered. Indeed, the personalization of the exercise protocol for estimating the individual perceived functional capacity shall be carried out by means of an activity-specific questionnaire, in conjunction with other baseline variables such as the determination of the sensor-based daily energy expenditure or cumulated walking time. Subjects should be interview via E-visit and telehealth for maintaining social distancing [3]. Motorized treadmill or a stationary cycle ergometer, among the vast spectrum of exercise modalities, are typically used for assessment of exercise capacity. However, cycle ergometry is preferred in elderly subjects because the ambulation instability may alter the results of the exercise test. Exercise tests with large increments of work rate have weaker correlation with oxygen uptake (VO_2_). Therefore, the Naughton [4] protocol, which only involve modest increases in work rate per stage, are recommended in COVID-19 elderly individuals. Moreover, intervals of 10 to 60 s between increments in work rate are typically recommended for ramp protocols. As previously mentioned, the exercise should be tailored to the individual capacity and to yield a fatigue-limited exercise duration of 8 to 12 min. The choice of treadmill exercise testing should be limited to COVID-19 elderly individuals with a very active lifestyle and no evidence of cardiovascular and pulmonary complications at rest. In this case, minimal or no handrail support should be encouraged, because this will otherwise reduce the work performed at any given level and alters the relationship between VO_2_ and work rate.

## Data acquirement during exercise

Heart rate, ECG, blood pressure and blood oxygen saturation should be monitored continuously throughout the exercise test (Fig. 1). Moreover, perceived exertion and symptoms (dyspnea/angina) should be recorded at regular intervals (60 s) throughout the exercise test. Dyspnea or angina should be recorded at symptoms onset with 1-to-4 scales. Due to the potential consequences of COVID-19 on cardiac function, the assessment of ECG and potential altered responses such as arrhythmias, ST-segment shifts are necessary. It is also important to screen the appearance of the elderly subject for identifying potential changes of alertness, coordination, and responsiveness during exercise. Termination criteria for exercise testing include subject’s request, moderate to severe dyspnea or angina, specific ECG changes (arrhythmias or ST-segment shifts), abnormal blood pressure responses and decrease in blood oxygen saturation > 4%. Monitoring the heart rate, blood pressure, ECG and blood oxygen saturation during recovery is important because abnormalities occurring during the post-exercise period provide valuable diagnostic and prognostic information.

## Emergency procedures

Although exercise testing (maximal and submaximal) is a safe procedure, even in the oldest-old and frail population, specific attention needs to be focused on potential COVID-19 sequels on cardiovascular and pulmonary systems. All testing facilities (clinical and subclinical) must have equipment, drugs, and personnel trained to deliver appropriate emergency care as previously reported in the joint position papers of the AHA and the ACSM [5]. A specific plan for rapid accessibility should be clearly defined when the extensive equipment cart is located in an area other than the testing area. A defibrillator should be always present in every exercise laboratory, and its function should be monitored on a daily basis and recorded in a log for quality control.

## References

[1] Schaller T, Hirschbuhl K, Burkhardt K, Braun G, Trepel M, Markl B, Claus R (2020). Postmortem examination of patients With COVID-19. JAMA.

[2] Hui DS, Wong KT, Ko FW, Tam LS, Chan DP, Woo J, Sung JJ (2005). The 1-year impact of severe acute respiratory syndrome on pulmonary function, exercise capacity, and quality of life in a cohort of survivors. Chest.

[3] Venturelli M, Cè E, Paneroni M, Guazzi M, Lippi G, Paoli A, Baldari C, Schena F, Esposito F (2020). Safety procedures for exercise testing in the scenario of COVID-19: a position statement of the Società Italiana Scienze Motorie e Sportive. Sport Sci Health.

[4] Naughton J, Balke B, Nagle F (1964). Refinements in method of evaluation and physical conditioning before and after myocardial infarction. Am J Cardiol.

[5] American College of Sports M, American Heart A (2002). American College of Sports Medicine and American Heart Association joint position statement: automated external defibrillators in health/fitness facilities. Med Sci Sports Exerc.

